# Robotic-assisted vs conventional surgery in medial unicompartmental knee arthroplasty: a clinical and radiological study

**DOI:** 10.1186/s43019-021-00087-2

**Published:** 2021-02-12

**Authors:** Roberto Negrín, Jaime Duboy, Magaly Iñiguez, Nicolás O. Reyes, Maximiliano Barahona, Gonzalo Ferrer, Carlos Infante, Nicolás Jabes

**Affiliations:** 1 Department of Orthopedics and Traumatology, Clinica Las Condes, Estoril 450, Las Condes, Santiago, Chile; 2 Department of Orthopaedic Surgery, Hospital Clinico de la Universidad de Chile, Santiago, Chile

**Keywords:** Unicompartmental knee, Robotic-assisted surgery

## Abstract

**Background:**

The use of unicompartmental knee arthroplasty (UKA) has increased and new technologies have been developed to improve patient survival and satisfaction, soft tissue balance, alignment, and component size. Robot-assisted systems offer an increase in surgical precision and accuracy. The purpose of this study is to evaluate the precision of component position using five radiological parameters in conventional and robotic-assisted medial UKA using the NAVIO system.

**Methods:**

A cohort study was designed for patients who underwent medial UKA between April 2017 and March 2019 in a single center. Patients were allocated in the conventional (UKA-C) or robotic-assisted (UKA-R) group. The variables analyzed were age, gender, affected knee side, length of hospital stay, surgical time, and radiological measurements such as anatomical medial distal femoral angle (aMDFA), anatomical medial proximal tibial angle (aMPTA), tibial slope, the sagittal femoral angle, and the component size. A target was defined for each measurement, and a successful UKA was defined if at least four radiological measures were on target after surgery. Also, patients’ reported outcomes were evaluated using the Oxford Knee Score (OKS) and a numeric rating scale (NRS) for pain.

**Results:**

Thirty-four patients were included, 18 of them underwent UKA-R. The success rate for UKA in the UKA-R group was 87%; meanwhile, in the UKA-C group this was 28%, this difference was significant and powered (Fisher’s exact test, *p* = 0.001; 1 − *β* = 0.95). Also, a 5-point difference in favor of the UKA-R group in the median OKS (*p* = 0.01), and a significantly lower median NRS for pain (*p* < 0.000) were found after surgery.

**Conclusions:**

UKA-R achieved more precision in the radiological parameters’ measure in this study. Also, UKA-R has a trend towards a better OKS and a lower NRS for pain at short-term follow-up.

## Background

Knee osteoarthritis is a prevalent disease affecting up to 19% of the population over 45 years of age, causing chronic pain, disability, and lower quality of life [1–8].

Unicompartmental knee arthroplasty (UKA) is a cost-effective treatment for femorotibial unicompartmental knee osteoarthritis. Over the last few years, the proportion of UKA had been increasing according to worldwide national registers [9–14]. Patients undergoing UKA had a lower rate of complications, faster functional recovery, and better satisfaction compared to total knee arthroplasty (TKA) [15–18]. Nevertheless, UKA has its difficulties. To achieve an adequate position of the components – crucial in avoiding early aseptic loosening – is more challenging than in TKA. Robotic assistance may play an essential role in decreasing the rate of malposition of the components in UKA [19, 20], theoretically leading to better clinical results and longer survival rates [21–27].

Few publications describe the radiological findings after robotic-assisted UKA using NAVIO (Blue Belt Technologies, Plymouth, MN, USA) [28]. This study aims to evaluate the precision of component position using five radiological parameters in conventional and robotic-assisted medial UKA using the NAVIO system. The hypothesis is that the robotic-assisted technique has a better rate of success compared to conventional UKA. Secondary aims were to compare short-term patient-reported outcomes and pain between groups and to analyze whether an adequate component position related to better short-term results in UKA.

## Methods

### Patient selection

A cohort study was designed for patients who underwent medial UKA between April 2017 and March 2019 in a single center. The local Ethical Committee approved the study, and all patients signed, written informed consent before enrollment.

All patients’ clinical records were reviewed, and their pre- and post-operative radiographs were measured. All patients undergoing medial UKA, having undergone a pre- and post-operative radiological study were included. Patients were excluded if they had an incomplete documentation or refused to participate.

Patients were allocated in the conventional (UKA-C) or robotic-assisted (UKA-R) group according to the surgeon’s and patient’s preference and the availability of the robot at the time of the surgery. In all patients, the Journey UNI implant (Smith & Nephew Inc., Cordova, TN, USA) was used. All procedures were performed under spinal anesthesia by two senior knee surgeons (JD, R.N.). UKA-C was defined as the surgery performed without robotic assistance, and UKA-R as the surgery performed with the assistance of the NAVIO Robotic System (Blue Belt Technologies, Plymouth, MN, USA). With the use of a tourniquet, an anterior approach and a medial parapatellar arthrotomy were performed in all surgeries. No wound drain was used.

The variables analyzed were age, gender, length of hospital stay, surgical time, and radiological measurements. The time of surgery in the UKA-C group ranges from incision to wound closure; meanwhile, in the UKA-R group, it was from the positioning of the pins (before the surgical incision) to wound closure.

### Radiological measurements

Radiological measurements were performed using the immediate post-operative radiographs. An anteroposterior (AP) and a lateral knee radiograph were performed in all patients on day 1 after surgery. Two blinded orthopedic surgeons (NR, NJ) carried out the measurements.

**Fig. 1:**
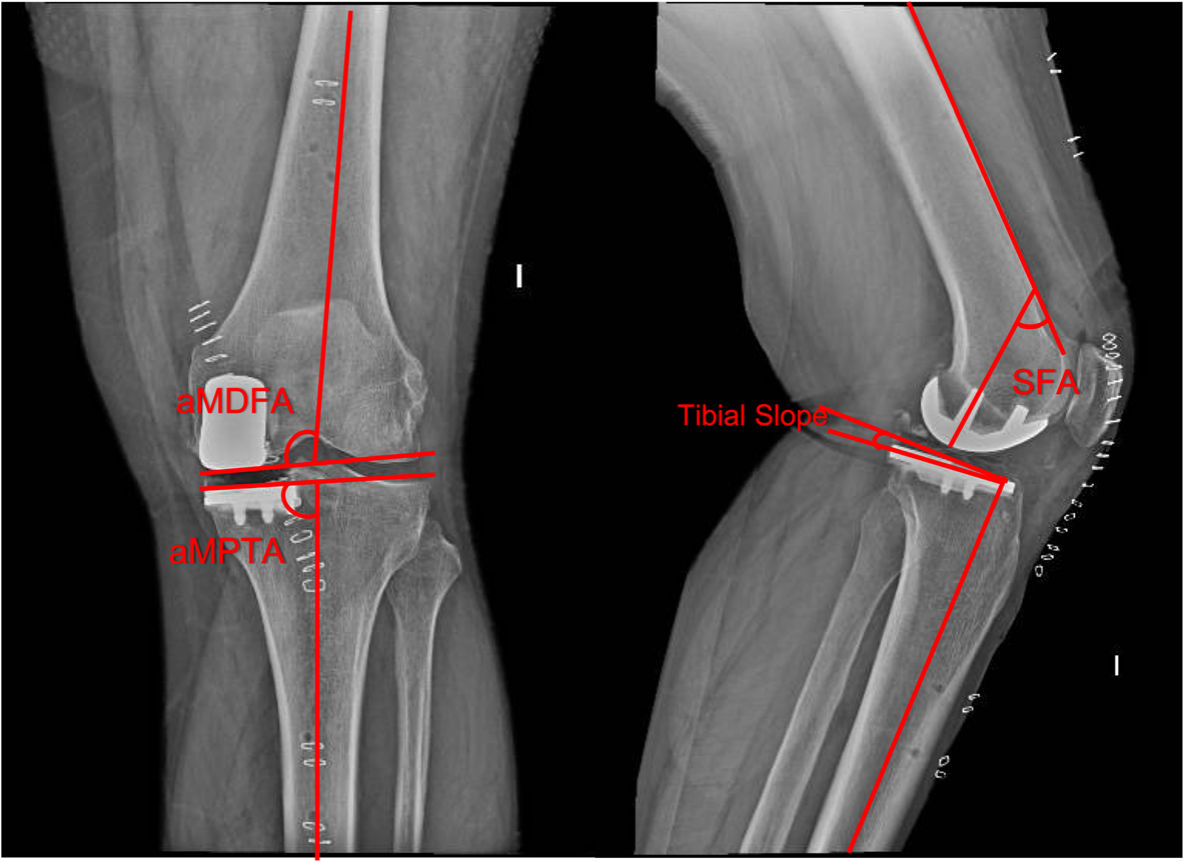
Radiological measures in the anteroposterior and lateral knee x-ray. *aMDFA* anatomical medial distal femoral angle, *aMPTA* anatomical medial proxial tibial angle, SFA sagital femoral angle

The same protocol used in the study by Iñiguez et al. [19] was used (Fig. 1). The anatomic medial distal femoral angle (aMDFA) was defined as the angle between a line through the anatomical axis of the femur and a line that joins the most distal point of the lateral condyle and the medial femoral component. The sagittal femoral angle (SFA) was defined as the angle between a line through the anterior cortex of the metaphyseal-diaphyseal junction and a line through the posterior peg of the femoral component.

The anatomical medial proximal tibial angle (aMPTA) was defined as the angle between a line through the anatomical axis of the tibia and a line through the tibial component. The tibial slope was defined as the angle between a line through the sagittal mechanical axis of the tibia and a line through the tibial component.

The femoral and tibial component size was assessed in the lateral knee radiograph, according to the designer classification: Oversized was defined when the implant protrudes more than 2 mm and undersized when the implant does not achieve adequate coverage. The target for each measurement was defined as follows: tibial slope 5 ± 3°, aMDFA 98 ± 3°, aMPTA 87 ± 3°, SFA 45 ± 3° and an adequate component size.

### Clinical measurements

All patients were evaluated pre-surgery, and then they were contacted 6 months after surgery by a blinded evaluator to request that they complete the Oxford Knee Score (OKS) and to evaluate knee pain at rest using a numeric rating scale (NRS) from 0 to 10, with 0 being no pain.

### Statistics

The frequency, proportions, median, range, and interquartile range were used to describe the sample of the study. The nonparametric median test was used to compare the continuous variables: age, affected knee side, length of hospital stay, surgery time, OKS, NRS, and radiographic measurements. Fisher’s exact test was used to compare categorical variables: gender, side, and component size. Multivariate logistic discrimination analysis was conducted to assess the capacity to recognize whether the UKA was robotic-assisted or conventional using the radiological parameters as predictor variables.

The frequency of success in each radiological measurement was compared between groups using a Fisher’s exact test. A successful UKA was considered if the target was achieved in at least four of the radiographic parameters. The proportion of successful UKAs was compared between groups using Fisher’s exact test. The power of the estimation (1 − *β*) was reported and considered underpowered if 1 − *β* was lower than 0.8. Also, to analyze the relevance of a successful UKA, a nonparametric median test was used to compare the median OKS and pain NRS between successful and failed UKAs.

Confidence intervals of ±95% were built, and a significance level of 0.05 was used. The data were processed using Stata 11.2 version (StataCorp LP, College Station, TX, USA).

## Results

A total of 34 patients were included, of whom 18 were UKA-R patients (Table 1). No significant differences were found between the two groups in terms of gender, age, affected knee side, or pre-operative OKS or pain NRS (Tables 1 and 2). Also, no differences were found in pre-operative radiological measurements. The median total surgery time was significantly lower in UKA-C patients (*p* < 0.01) (Table 2).

**Table 1:** Comparison of demographics variables between groups

	UKA-R	UKA-C	Total	***P***
***N***	16 (47%)	18 (53%)	34	N/A
**Male**	7 (44%)	12 (67%)	19 (56%)	0.16*
**Age**	66 (56 to 82)	65 (41 to 76)	66 (41 to 82)	0.60**
**Right side**	10 (63%)	8 (44%)	16 (47%)	0.32*

*UKA-R* robotic-assisted unicompartmental knee arthroplasty *UKA-C* conventional unicompartmental knee arthroplasty, *N* number

*Fisher’s exact test, **nonparametric test median

**Table 2:** Comparison of clinical and radiological measurements between groups

	UKA-R	UKA-C	***P***
**BS OKS**	17 (6–41) [11–23]	18 (12–30) [15–21]	0.34**
**BS Pain NRS**	6 (2–10) [5–7]	7 (2–10) [6–8]	0.52**
**Lengh of hospital stay (days)**	2 (1–4) [2–3]	2 (1–3) [2–2]	0.25**
**Surgery time**	139 (125–156) [129–72]	106 (85–131) [98–124]	< 0.01**+
**Sagital femoral angle**	46 (42–57) [44–47]	48 (33–65) [44–53]	0.47**
a**MDFA**	98 (95–101) [97–99]	101 (96–105) [98–103]	0.02**+
a**MPTA**	86.9 (83–92) [85–88]	85.3 (81–97) [83–86]	0.26**
**Slope**	4.4 (1.7–7.0) [3.3–5.1]	5.3 (0.7–10.0) [3.8–6.9]	0.09**
**Incorrect size**	0 (0%)	4 (22%)	0.07*
**Post-operative OKS**	45 (37–47) [41–47]	39 (23–48) [37–42]	0.01**+
**Post-operative pain NRS**	1 (0–3) [0–1]	4 (0–9) [2–6]	< 0.01**+

*UKA-R* robotic-assisted unicompartmental knee arthroplasty, *UKA-C* conventional unicompartmental knee arthroplasty, *BS* before surgery, *NRS* numerical rating scale, *aMDFA* anatomical medial distal femoral angle, *aMPTA* anatomical medial proximal tibial angle, *OKS* Oxford Knee Score

*Fisher’s exact test; **nonparametric test median; + significant difference

The post-operative radiographic measurements are summarized in Table 2. Only the SFA reached a significant difference (nonparametric median test, *p* = 0.02) between groups. The ability to discriminate between groups using the radiological parameters was high, being the error classification error 0.11. Only one UKA-R was misclassified as UKA-C, but two UKA-Cs were misclassified as UKA-R.

The rate of success to achieve the desired target in each radiological measurement is summarized in Table 3. Successful UKA was achieved in 14 (88%) of the UKA-R patients, but only five (28%) of the UKA-C patients (Table 4). The proportion of successful outcomes was statistically significant and powered (Fisher exact test, *p* = 0.001; 1 − *β* = 0.95).

**Table 3:** Frequency that each group achieved the desired target in radiological parameters

	UKA-R	UKA-C	P*
a**MDFA 98** **± 3°**	14 (88%)	10 (56%)	0.06
a**MPTA 87 ± 3°**	13 (81%)	11 (61%)	0.27
**Tibial slope 5 ± 3°**	15 (94%)	15 (83%)	0.60
**Sagittal femoral angle 45 ± 3°**	12 (75%)	5 (28%)	0.02+
**Correct implant size**	16 (100%)	14 (78%)	0.11

*UKA-R* robotic-assisted unicompartmental knee arthroplasty, *UKA-C* conventional unicompartmental knee arthroplasty, *aMDFA* anatomical medial distal femoral angle, *aMPTA* anatomical medial proximal tibial angle

*Fisher’s exact test, + significant difference

**Table 4:** The number of desired targets achieved by each patient who underwent UKA. Successful UKA was defined when at least four radiological parameters reached the desired goal. UKA-C achieved a success rate of 0.28; meanwhile, UKA-R achieved a rate of 0.88. The proportion of successful outcomes was statistically significant and powered (Fisher’s exact test, *p* = 0.001; 1 − *β* = 0.95)

***N***° of target achieved	UKA-R	UKA-C
**5**	9 (56%)	3 (17%)
**4**	5 (31%)	2 (11%)
**3**	2 (13%)	8 (44%)
**2**	0	4 (22%)
**1**	0	1 (06%)
**0**	0	0

*UKA-R* robotic-assisted unicompartmental knee arthroplasty, *UKA-C* conventional unicompartmental knee arthroplasty, *N* number

***N***° Number of targets achieved

**Fig. 2:**
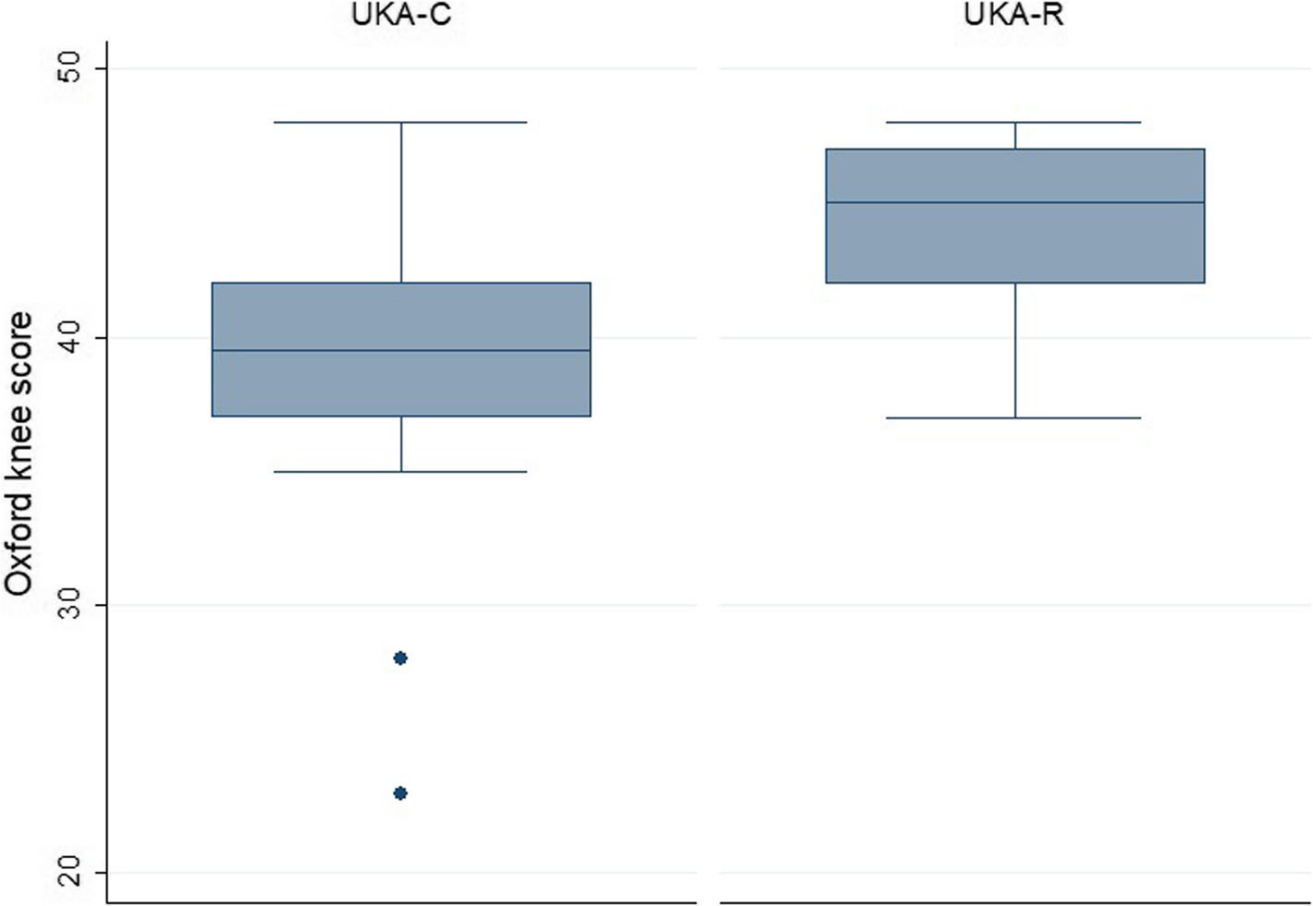
Distribution of Oxford Knee Score (OKS) among groups. Robotic-assisted unicompartmental knee arthroplasty (UKA-R) had a trend to better OKS than conventional unicompartmental knee arthroplasty (UKA-C) (nonparametric median test, *p* = 0.01)

**Fig. 3:**
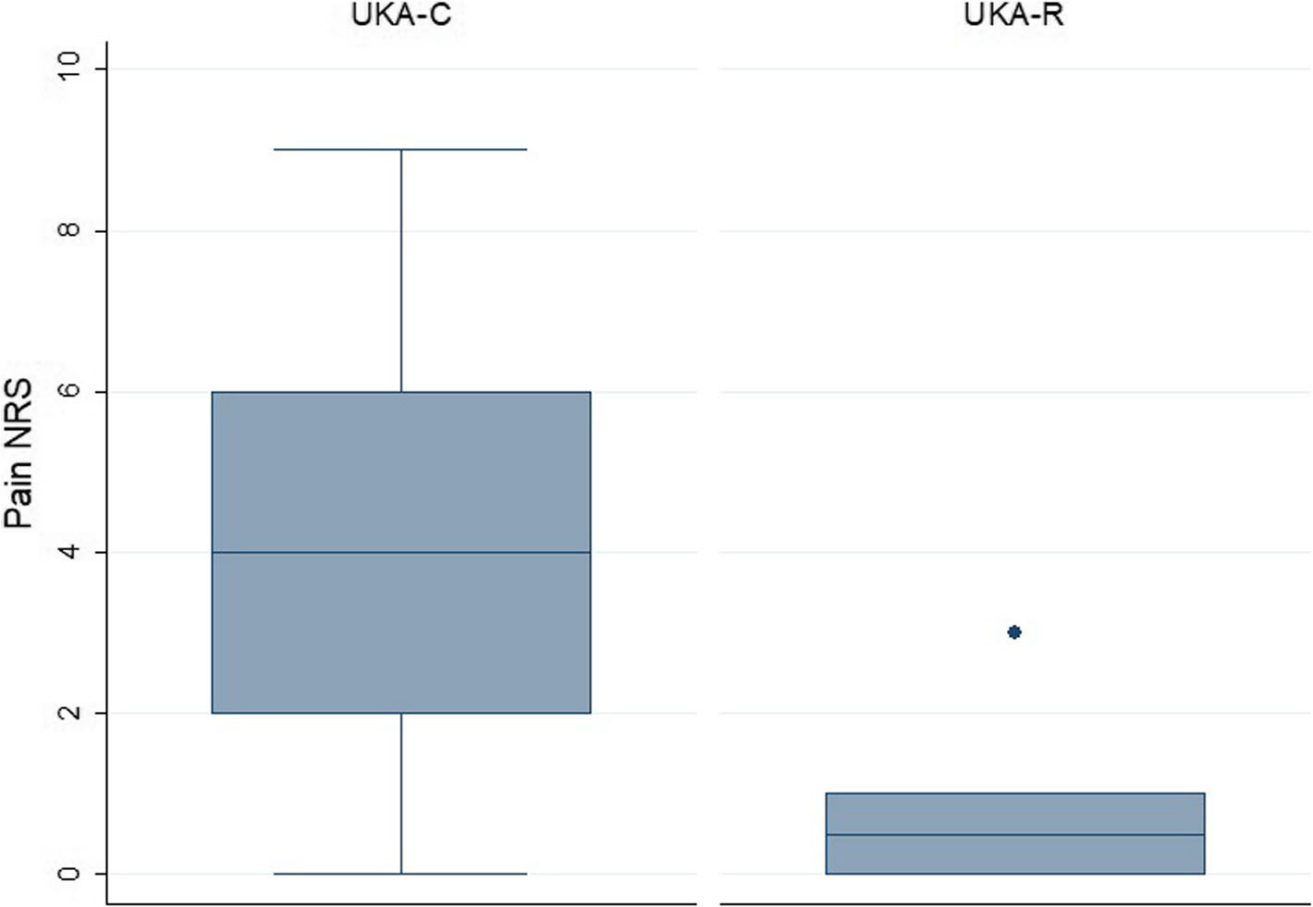
Distribution of numerical rating scale (NRS) for pain among groups. Robotic-assisted unicompartmental knee arthroplasty (UKA-R) had a trend to lower NRS for pain than conventional unicompartmental knee arthroplasty (UKA-C) (nonparametric median test, *p* < 0.000)

A significantly better OKS was achieved in the UKA-R group (nonparametric median test, *p* = 0.01) (Fig. 2). Yet, the median difference between groups was only 5 points, so it may not be clinically relevant (Table 2). The median pain NRS in the UKA-R was 1 (range, 0 to 3) and 4 in the UKA-C (Fig. 3); this difference reached statistical significance (nonparametric median test, *p* < 0.000).

Successful UKA had a trend towards a higher OKS (nonparametric median test, *p* = 0.088; 1 − *β* = 0.51) and a significantly lower pain NRS (nonparametric median test, *p* = 0.036; 1 − *β* = 0.57), but both findings were underpowered.

There was only one complication in the UKA-C group; this was an arthrofibrosis that required mobilization under anesthesia. No complications were found in the UKA-R group.

## Discussion

The main finding of this study is that UKA-R yielded a significantly higher rate of radiological successful UKA. Also, there was a trend towards a better functional patient-reported outcome and less pain during rest. Moreover, the definition of successful UKA by the radiological measurements used in this study was significantly related to better functional outcomes and lower pain levels during rest, yet this finding was underpowered. This study is a continuation of the clinical phase in the cadaveric pilot study published by our team, which showed consistent results of greater accuracy in relation to implant position in robotic-assisted surgery, compared to the conventional technique [19].

Published studies have shown that robotic-assisted surgery allows for greater accuracy and improved implant position [29–33].

In the radiological assessment of the cases studied, we considered five imaging parameters and we defined success as the fulfillment of at least four out of five of the requirements since we believe that combining all the radiological parameters achieved a more comprehensive way of measuring accuracy. We have no references to previous publications in which this way of assessing accuracy is used, and considering the prosthesis as a whole and not analyzing each radiological variable in particular seem to provide a more comprehensive evaluation.

By analyzing the results of the radiological measurements in both groups separately, there are only statistically significant differences among them in terms of SFA. However, when considering the objective of success with the predefined parameters, the robotic group reaches 88% versus 28% for the conventional group (*p* = 0.001). This correlates with the logistic discriminant analysis of both groups, which shows an error rate of 0.11, with only one UKA-R wrongly categorized as conventional. We defined an interval of ±3° as it was the interval that maximized the difference between the groups. Bell et al. published a difference with an interval of ±2° [34]; however, in our study using an interval of ±1° more in width we found a significant difference.

The first published data on the accuracy of implant positioning using the NAVIO system were promising. Batailler et al. published the first clinical study comparing robotic-assisted UKA versus the conventional technique [24]. The authors conclude that there is a significant improvement in the accuracy of implant positioning with robotic-assisted surgery in the coronal as well as in the sagittal plane, thus reducing the number of outliers, but no significant difference was found in functional results among the groups studied. Our results were similar to those published by Batallier et al. regarding the accuracy of implant positioning, although we did find better functional and post-operative pain results in the UKA-R group compared to the UKA-C group at 1-year follow-up [24], which was similar to results published in other series [20] of robotic-assisted surgery.

In this series, we did not find any complications in either group, with just one case of manipulation under anesthesia in the conventional group that could explain the difference in the OKS and NRS in favor of the robotic group. This is different from what St. Mart reported; in his study he had a higher rate of revision in the robotic group because of early infection [35]. The fact that we did not find any complications in the robotic group makes us believe that this is a safe procedure, even though these were our first cases, which was similar to what Mergenthaler [36] published in his case-control series.

The limitations of this study include its small sample size and short-term follow-up of 6 months, and not the 2-year follow-up that most studies report, which prevented us from adequately demonstrating some of the trends observed and does not necessarily represent long-term differences. There also may be a bias for the pain NRS score, and this may explain why the results of the NRS in conventional surgery are higher than those reported in the literature. Another limitation is the way that accuracy is measured with x-rays, which does not allow us to assess the rotational effects on implants. In the study design, we rejected the use of computed tomgraphy scans due to the need for patient exposure to radiation which is equivalent to 48 chest x-rays, as Ponzio et al. [37] pointed out, and also due to the high cost associated with it, cost being one of the advantages of the imageless robotic NAVIO system used.

Future studies in larger population groups and long-term follow-ups are necessary to confirm the trend observed in the favorable results of our study.

## Conclusions

Robotic-assisted UKA with the NAVIO system offers greater accuracy of femoral implant positioning in the sagittal plane, and it is more accurate in achieving clinical and radiological success compared to conventional surgery.

## Data Availability

All data generated or analyzed during this study are included in this published article and its supplementary information files.
